# 

**Published:** 1844-12

**Authors:** 


Page

### LIBRARY.

A new Treatise on the Theory and Practice of Dental Surgery. By J. Lefoulon, Surgeon Dentist, Paris. Translated from the French, by Thomas E. Bond, Jr., M. D.................... 81

### JOURNAL.

ARTICLE I.—Contributions to Operative and Mechanical Dentistry. By W. H. Elliot, D. D. S.,.................................... 83
ARTICLE II.—Opening Address. By Jas. Taylor, M. D., D. D. S., 91
ARTICLE III.—Address, delivered before the Mississippi Valley Association of Dental Surgeons. By Dr. John Allen,..... .... 105
ARTICLE IV.—Proceedings of a Convention of Professional Dentists. Held August 13th and 14th, 1844, in Cincinnati,............ 112
ARTICLE V.—Transactions of the Virginia Society of Surgeon Dentists. 120
ARTICLE VI.—Obituary. By George G. Brewster, D. D. S.,...... 123
ARTICLE VII.—Low Fees and Cheap Stock. By G. G. Brewster, M. D., ................................................ 126
ARTICLE VIII.—On the Use of Arsenic in Teeth. By Hudson S. Burr, M. D.,.......................................... ....... 127
ARTICLE IX.—On the Use of Arsenic in destroying the sensibility of a Carious Tooth, preparatory to Filling. By Edward Taylor, M. D., ............................................... 129
ARTICLE X.—Letter from Dr. A Van Camp,.................... 131
ARTICLE XI.—Address. By Dr. James Taylor, M. D., D. D. S., 133
ARTICLE XII.—Operation for Correcting Protrusion of the Under Jaw. By the Syracuse Editor,............................ 147

### COLLECTANEA.

The Necessity of appointing Professed Dentists to all public Hospitals and Dispensaries,........................................ 152
Irregular and Decayed Teeth,.................................... 156
Health and Fecundity of the Region around Fort Kent, Maine,.... 157
Medical Lectures,.. . ......................................... 158
Multiplication of Dentists,...................................... 158
Drawing Teeth in Africa,............... ....................... 158

### BIBLIOGRAPHICAL NOTICES.

Dictionary of Practical Medicine. By Jas. Copland, M. D., F. R. S., 159

The Cyclopedia of Practical Medicine,............................ 159

### MISCELLANEOUS NOTICES.

Explanation,.................................................... 160
Gosslp with our Profession,...................................... 161
Obituary, Dr. John N. Frink,..................................... 162

